# Physicians’ lack of knowledge - a possible reason for red blood cell transfusion overuse?

**DOI:** 10.1186/s13584-017-0173-0

**Published:** 2017-12-12

**Authors:** Roni Rahav Koren, Celia Suriu, Orly Yakir, Luiza Akria, Masad Barhoum, Andrei Braester

**Affiliations:** 1 Azrieli Faculty of Medicine in the Galilee, Bar Ilan University, 8 Henrietta Szold St., 1589 Tzfat, Israel; 2 Galilee Medical Center, Nahariya, Israel; 3 Meir Medical Center, Kfar Saba, Israel

**Keywords:** Red blood cell transfusion, Knowledge, Medical overuse

## Abstract

**Background:**

A significant percentage of red blood cell transfusions are inappropriately overused. This study investigated physicians from the western Galilee in terms of their knowledge of transfusion medicine as a potential reason for red blood cell overuse, and assessed the influence of personal background characteristics on their knowledge.

**Methods:**

Data were collected via anonymous questionnaires. The questionnaires included a personal background section and a professional section. Study participants were grouped according to field of specialty, seniority, and location of medical school graduation, in order to correlate participant characteristics with knowledge.

**Results:**

Scores were calculated on a 0–100 scale. The overall knowledge of the study population was low (mean score 47.8 ± 18.6). Knowledge regarding basic physiology of red blood cell transfusion was also low. Internal medicine physicians and senior physicians had significantly greater overall knowledge scores and were more familiar with a restrictive blood management policy than were surgeons and residents, respectively. Comparing knowledge scores, no difference was found regarding indications for transfusion.

**Conclusion:**

General and fundamental knowledge in transfusion medicine is lacking among physicians in the non-operating room setting, which may play a role in red blood cell transfusion overuse. Field of specialty and professional status influenced knowledge of transfusion medicine. Educational programs and increased physicians’ awareness might help decrease unnecessary transfusions.

**Trial registration:**

Not applicable.

**Electronic supplementary material:**

The online version of this article (10.1186/s13584-017-0173-0) contains supplementary material, which is available to authorized users.

## Background

Despite the risks and high costs associated with red blood cell (RBC) transfusion (annual expenditure of $1.62 to $6.03 million per hospital in the United States and Europe) [1, 2], the Joint Commission along with the American Medical Association has included blood transfusions in a list of the five most overused therapeutic procedures in the United States [3], where 15 million blood units are given per annum (one unit every 0.5 s). In Israel, according to the national Israeli blood bank spokesperson, 522,000 blood products were sold to the Israeli hospitals in 2013, and approximately 350,000 RBC units are sold each year. According to US and Israeli demographics in 2013, a similar number of 0.04 RBC units was given per person.

For many decades, the decision to transfuse RBCs followed a liberal approach, which was to maintain blood hemoglobin concentration above 10 g/dl. Reevaluation of this threshold trigger raised fundamental issues regarding its arbitrariness, as well as a lack of evidence [4, 5] for the basis of many aspects of transfusion practice, when compared with other fields of medicine.

Hence, a growing number of international studies comparing a restrictive blood management approach (using a lower hemoglobin transfusion threshold of 7–8 g/dl) to the previously prevailing liberal approach (using the 10 g/dl threshold) has been generated [6–11]. A landmark study among intensive care unit patients established by the Canadian Critical Care Trials group (TRICC trial), showed that a restrictive RBC transfusion strategy is at least as effective as and possibly superior to a liberal transfusion strategy for critically ill patients (with the possible exclusion of patients with acute coronary syndrome) [6].

A Cochrane meta-analysis suggested that compared with a target hemoglobin of 10 g/dl, hemoglobin target values of 7 to 8 g/dl are associated with equivalent or better outcomes [12].

Another study published by Stanford University Medical Center [13] assessed patient outcomes before and after implementation of real-time clinical decision support for transfusion when hemoglobin level was 7 to 8 g/dl. The study compared patient outcomes (mortality, length of hospital stay and 30-day readmission rate) hospital-wide before implementation of the clinical decision support (January 2008 to July 2010) and after (July 2010 to December 2013). This study concluded that improved blood utilization with the restrictive blood management approach was associated with stable or improved outcomes and total savings in acquisition costs of approximately $6.4 million.

In 2012, AABB established an evidence-based guideline [14] with specific transfusion thresholds regarding hemodynamically stable adult and pediatric, medical and surgical patients in order to standardize transfusion practice. Furthermore, the Choosing Wisely campaign, which promotes the appropriate use of health care resources, calls for limiting transfusions and lowering the transfusion threshold according to the AABB recommendations [15]. Nevertheless, the global waste of blood products is still overwhelming, and if a restrictive transfusion strategy was widely implemented to replace a liberal strategy, exposure of patients to red blood cell transfusions would decrease by an average of approximately 40% [14].

Amongst the numerous potential reasons for RBC transfusion over-utilization, with the exception of physicians who practice transfusion medicine, and in the non-operating room setting, we believe that physicians’ lack of fundamental knowledge in the field of transfusion medicine may play an important role.

This study investigated the knowledge of transfusion medicine among physicians in the surgical and internal medicine departments at the Galilee Medical Center. Physicians completed a questionnaire investigating familiarity with the discipline of restrictive blood management, as well as indications for blood transfusion.

By classifying the study population according to personal background including age, medical school, medical specialty, and professional status, we evaluated the influence of these factors on physicians’ knowledge regarding transfusion medicine.

Considering that physicians’ lack of knowledge is a potential reason for RBC overuse, we assumed that the general knowledge of the study population would be low (less than 50% correct answers). Highlighting this issue could promote attempts to seek educational tools, increase physicians’ awareness, and encourage the Israeli Ministry of Health to reduce unnecessary transfusions.

## Methods

### Participants

Among 141 physicians who were listed as manpower in the Galilee Medical Center, 53 were not available during the meetings in which the questionnaire was administered (some were on maternity leave, were no longer employees, were abroad, or were on rotation). Thus, 79 physicians from seven internal medicine and surgical departments (general surgery, orthopedics, obstetrics & gynecology and urology) completed the questionnaire. The overall response rate was 56% and 90% of physicians who were available to participate completed the questionnaire. All were employed at Galilee Medical Center during 2014.

The Galilee Medical Center is a tertiary medical center with 710 beds. It serves the majority of the population of 600,000 in northern Israel. In the year prior to the study, annual RBC consumption hospital-wide was 6500 units.

Participants were classified into the following groups in order to correlate participant characteristics with knowledge: internal medicine physicians vs. surgeons, residents vs. senior physicians, and graduate of a medical school in Israel vs. another country. In order to create a more closely matched comparison, knowledge of physicians in the non-operating room setting was examined.

Sample size calculation was based on data from a pilot study that included 9 completed questionnaires, demonstrating mean overall knowledge score (the mean of all scores, multiplied by 100) of 40 ± 20. A sample of 50 physicians provided a confidence of the mean of CI 95% (34, 46) with alpha set at 5%.

### Study design

Data were collected using a questionnaire (see Additional file 1) which was based on the AABB guideline [14]. Previous studies assessing knowledge of transfusion medicine only examined specific groups of physicians, (e.g. seniors, residents, post-graduate year 1 physicians) and the questionnaires were designed accordingly. Because we aimed to investigate physicians’ knowledge with an emphasis on knowledge and familiarity with a restrictive policy, the AABB guideline seemed most applicable to generate the study questionnaire. The questionnaire was designed and formulated by the investigating team, including the institutional blood bank director. The questionnaire was validated by peer review of the three hematologists at our institute with the greatest interest in transfusion medicine. Following selection of the topics, questions were formulated to address basic knowledge of the physiology of blood transfusion, familiarity with restrictive blood management, and clinical indications for RBC transfusion. A pilot study was performed using 9 completed questionnaires in order to demonstrate the questionnaire’s validity and reliability. The Cronbach alpha coefficient for reliability measurement was α = 0.7.

The questionnaire was composed of a personal background section (8 questions), and a professional section (20 questions). The professional section examined general knowledge in transfusion medicine (evaluated as the overall questionnaire), familiarity with restrictive blood management discipline (evaluated by eight questions: 1, 3–6, 10, and 19–20) and knowledge regarding indications for transfusion (evaluated by six questions: 9, 11–15). Individual scores were calculated based on the proportion of correct answers for every subject that was examined. Each correct answer received one point and an incorrect answer zero points. The sum of all correct answers was divided by the number of relevant questions included for each subject and was multiplied by 100 (total scores ranged from 0 to 100). A higher score was given to two questions about the basic physiology of transfusion (7 and 8) that were considered particularly basic, based on consensus of both the investigators and the reviewers that all physicians at all training levels should be able to answer these questions correctly.

The questionnaires were anonymous and were given to and collected from the physicians directly by the researcher during morning meetings, from February 2014 through March 2014.

### Data analysis

Continuous data were described by means, standard deviations, quartiles (median, interquartile range (IQR)) and ranges. Categorical data were analyzed using Chi-square test or Fisher’s exact test, according to the test’s assumption and were described by frequencies and percentages. Answers were defined as new dichotomous variables (correct/incorrect), which were then calculated as the proportion of the correct answers or summed appropriately.

### Univariate analysis

Analysis of variance (ANOVA), independent sample *t*-test or Wilcoxon rank sum test were used to compare quantitative data among groups. The tests were chosen according to distribution of the variables and according to the number of groups compared.

### Multivariate analysis

Multivariate linear regression models were used to examine general knowledge, defined as the mean scores of the overall questionnaire and the familiarity with restrictive blood management, controlling for field of specialty, professional status and place of medical school graduation.

## Results

***Sample description*** is shown in Table 1. The mean age of the study population was 40 years, 76% were men and 23% were women. Forty-three percent were specialists in internal medicine and 56% were surgeons. Among these, 40.5% were senior physicians and 59.5% were residents. Mean seniority was 12.8 years. Regarding place of medical studies, 24% graduated in Israel and 60% had graduated outside Israel, with the highest rate (30.3%) in the Former Soviet Union. Place of graduation was not available for 16.5% of the population study.

**Table 1:** Personal background of population study (*N* = 79)

Demographic characteristic		Number of responses
Gender	Male	60 (75.9%)
	Female	18 (22.7%)
	NA	1 (1.2%)
Age range (years)	Mean ± SD	39.9 ± 10.4
	Range	26–66
	25–34	33 (41.7%)
	35–44	17 (21.5%)
	45+	25 (31.6%)
	NA	4 (5.0%)
Professional status	Senior physician	32 (40.5%)
	Resident	47 (59.5%)
Seniority (years)	Mean ± SD	12.8 ± 10.93
	Median	9.0
	Range	0.25–40.0
Medical specialty	Internal medicine	34 (43.0%)
	Surgery	44 (55.7%)
	NA	1 (1.2%)
Place of graduation	Israel	19 (24.0%)
	Other country	47 (59.5%)
	NA	13 (16.5%)

*SD* standard deviation, *NA* no answer

**Fig. 1:**
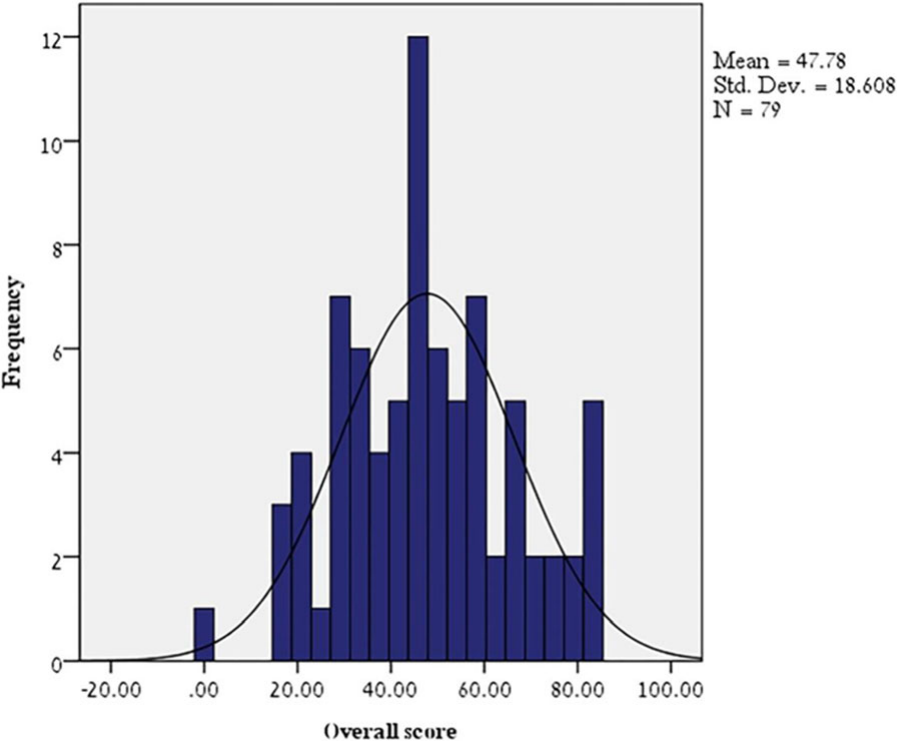
Scores of the overall questionnaire. Distribution of the overall knowledge scores of the study population. Mean overall knowledge score of the population study was 47.8 ± 18.6

***Scores of the overall questionnaire*** results are depicted in Fig. 1. The mean score of the population study was 47.8 ± 18.6.

### The influence of personal background on knowledge of transfusion medicine

Mean scores were calculated and compared according to personal background groups and are depicted in Table 2 **.** Internal medicine and senior physicians had significantly greater overall knowledge than did surgeons and residents, respectively (mean scores: 55 for internal medicine physicians vs. 42 for surgeons, *P* = 0.02 and 54 for seniors vs. 43 for residents, *P* = 0.01). No difference was found when comparing place of graduation (*P* = .37).

**Table 2:** The influence of personal background on knowledge of transfusion medicine

Variable		Overall score (0–100)		Familiarity with the restrictive blood management discipline score (0–100)		Indications for transfusion score (0–100)	
		Mean ± SD (IQR)	*P*-value (2-sided)	Mean ± SD (IQR)	*P*-value (2-sided)	Mean ± SD (IQR)	*P*-value (2-sided)
Age (Years)	25–34	43.8 ± 15.5 (29.1–54.1)	0.05^b^	43.3 ± 27.8 (20.0–65.0)	0.1^b^	49.4 ± 22.6 (33.3–66.6)	0.3^b^
	35–44	46.0 ± 21.0 (37.5–58.3)		51.7 ± 30.0 (25.0–80.0)		38.2 ± 27.4 (16.6–58.3)	
	45+	55.0 ± 17.4 (41.6–68.7)		58.8 ± 24.5 (40.0–80.0)		44.6 ± 24.8 (16.6–66.6)	
Professional status	Senior physicians	54.1 ± 19.5 (41.6–69.7)	0.01^a^	61.2 ± 26.6 (42.5–80.0)	0.003^a^	43.2 ± 26.3 (16.6–66.6)	0.75^a^
	Residents	43.4 ± 16.7 (29.1–54.1)		42.3 ± 26.7 (20.0–60.0)		45.0 ± 24.5 (33.3–66.6)	
Place of graduation	Israel graduates	50.4 ± 18.9 (37.5–66.6)	0.73^a^	54.2 ± 28.5 (30.0–80.0)	0.62^a^	51.7 ± 17.4 (33.3–66.6)	0.2^c^
	Non-Israel graduates	48.7 ± 18.0 (33.3–58.3)		50.4 ± 28.6 (30.0–80.0)		44.3 ± 27.6 (16.6–66.6)	
Field of specialty	Internal medicine	55.5 ± 20.0 (33.3–71.8)	0.002^a^	60.5 ± 30.3 (30.0–90.0)	0.03^a^	49.5 ± 26.7 (29.1–70.8)	0.12^a^
	Surgical	41.8 ± 15.3 (33.3–50.8)		41.3 ± 23.4 (22.5–60.0)		40.5 ± 23.6 (33.3–50.0)	

*SD* standard deviation

^a^Independent sample t-test; ^b^Anova; ^c^Wilcoxon rank sum test

Knowledge regarding familiarity with the restrictive blood management demonstrated similar results (mean scores: 60 for internal medicine physicians vs. 41 for surgeons, *P* = 0.03 and 61 for seniors vs. 42 for residents, *P* = 0.03). It was also shown that overall knowledge and knowledge regarding familiarity with restrictive blood management increased with the respondent’s age. This finding correlates with the mean age groups according to professional status, where seniors were 50.2 ± 7.6 years of age and residents age 33.7 ± 6.0 years (*P* < 0.001).

No difference was found in mean knowledge scores among groups regarding indications for transfusion.

In multivariate analysis, a significant difference in the overall knowledge score was found in favor of internal medicine physicians vs. surgeons (*P* < 0.001) and senior physicians vs. residents (*P* = 0.005). Similar results were demonstrated regarding familiarity with the restrictive blood management discipline in the favor of internal medicine and senior physicians over surgeons and residents, respectively (*P* = 0.001, *P* = 0.004).

***Knowledge regarding physiologic reasons for transfusion*** was examined in questions 7 and 8, professional section. Both questions were answered correctly by 9%. In question 7, physicians were asked to state “TRUE OR FALSE” regarding whether the only reason to transfuse RBCs is to improve oxygen delivery. Physicians who answered FALSE were also asked to mention other reasons, if any, for RBCs transfusion other than to improve oxygen delivery; 53% answered FALSE and 30% mentioned volume related reasons.

***Knowledge about guidelines*** was examined in question 18 (Professional section). Physicians were asked to state TRUE OR FALSE regarding whether an absence of clear guidelines leads to confusion among physicians regarding RBC transfusion; 63% agreed.

## Discussion

RBC transfusion is a common therapeutic intervention with considerable variation in clinical practice. It has been included as one of the five most over-utilized therapeutic procedures in the United States [3]. Nevertheless, a substantial number of randomized, controlled trials support a restrictive transfusion strategy rather than a liberal approach in various patient populations [6–11].

We believe that in the non-operating room setting, physicians who do not practice transfusion medicine lack fundamental knowledge in this field, which may be a possible reason for RBC overuse. Our study was primarily aimed to assess physician knowledge about transfusion medicine as it related to participant characteristics.

The overall knowledge of the participating physicians was low (mean score < 50 on a 0–100 scale). A substantial number of respondents mentioned volume-related reasons for RBC transfusion, which suggests a lack of basic knowledge of the physiology of RBC transfusion.

Studying the influence of professional status on a physician’s knowledge showed differences in overall knowledge and familiarity with restrictive blood management, in the favor of senior physicians over residents. These results were contrary to what we expected, as we assumed that residents are influenced more by textbooks and guidelines, and less by habitual practice that we attributed more to seniors. Interestingly, regarding knowledge of practical indications for transfusion, residents scored slightly higher than senior physicians did, although this was not clinically or statistically significant.

Similarly, internal medicine physicians scored higher overall knowledge and were more familiar with restrictive blood management policy than surgeons were. These results were also contrary to our expectations, as we did not expect to find any difference in knowledge associated with field of medical specialty. After our study was conducted, Revel-Vilk et al. performed a cross-sectional survey on the number of RBC transfusions given in surgical and non-surgical departments with the highest volume of RBC use [16]. While the majority of RBCs were given in the non-surgical departments, “off-protocol” RBC transfusion (patients receiving > 1 RBC unit consecutively or transfusion given to non-bleeding non-active, cardiac patients with hemoglobin levels ≥ 8 g/dl) was more common in the surgical departments. This difference can be explained by the influence of the clinical policy, which can differ between internal medicine and surgery departments. The difference in the urgency of the clinical scenario between perioperative and general medical settings clarifies the need for improved multidisciplinary communication in relation to perioperative blood transfusion. As expected, no difference was found in knowledge regarding transfusion medicine based on country of graduation from medical school.

When asked about the existence of guidelines, 63% of respondents agreed that lack of clear guidelines is a source of confusion among physicians regarding RBC transfusion. Revel-Vilk et al. also agreed that there is a need for clear guidelines to facilitate wise transfusion-related choices [16].

Although Israeli guidelines do not exist at present, there are numerous other RBC transfusion guidelines [14, 17–23]. Most agree that RBC transfusion is unnecessary above hemoglobin of 10 g/dl and the lower trigger level varies between 6 and 8 g/dl. However, the use or indication for blood transfusion, packed cells, or blood products is not always based on a rigid set of indications. Clinical factors can also influence the decision to transfuse blood, packed cells or blood products. This indicates that practice, depending on the clinical scenario, does not always reflect knowledge.

The Stanford University Medical Center [13] has been able to reduce RBC transfusions significantly through implementing real-time clinical decision support using an interruptive alert with each RBC order. The alert contained transfusion guidelines, a link to relevant literature and a reason for transfusion. This clinical decision support was implemented following one year of education about transfusion guidelines via electronic communication and in-person meetings.

The Stanford University medical center study shows that an educative tool can be used to reduce unnecessary transfusions. This supports our assumption that physicians’ lack of knowledge has a major contribution on RBC overuse.

Following this study, the director of the blood bank at our institution initiated an education program that aims to increase physicians’ awareness, with a special focusing on restrictive blood management policy. The program included a series of lectures, specific clinical cases discussed in group meetings, international biennial conventions hosted by the institute, and a blood coordinator operating 24/7. In the year following the questionnaire, RBC utilization decreased to 4000 units hospital wide (approximately 40%) with a total cost savings of approximately 900,000 ILS.

Previous transfusion medicine assessments have been published, demonstrating deficits in physicians’ knowledge [24–26]. O’Brien et al. assessed the knowledge of post graduate year 1 physicians using a transfusion consent scenario and a written quiz [25]. Marked knowledge deficits were demonstrated, with scores ranging from 24% to 67%, with a mean score of 39%. In the largest international assessment, Haspel et al. assessed the knowledge of internal medicine residents at different stages of residency [26]. They found that internal medicine residents have poor knowledge of transfusion medicine, with an overall mean score of 46%. Gharehbaghian et al. examined the knowledge of senior physicians using a 50-question survey, and compared generalists to specialists in transfusion medicine (anesthesiologists, hematologists, oncologists and surgeons) [24]. The mean of correct answers was 33% and was considered one-third lower than expected.

To the best of our knowledge, trials assessing knowledge in transfusion medicine among Israeli physicians currently do not exist. Our study also examined physicians’ knowledge of selected groups at different training levels, different fields of specialty and different (international) places of medical school graduation and compared them in order to correlate participant characteristics with knowledge, to potentially target specific groups among our hospital’s clinicians who require additional training. Finally, this study suggests the results of testing transfusion medicine knowledge and a measure of practice prior to the assessment and 1 year following the assessment, during which an education program focusing especially on the restrictive policy was implemented. This measure reinforces our hypothesis.

Despite its heterogeneity, the composition of the study population may be a limitation as it was composed exclusively of physicians from Galilee Medical Center and mostly men and might not represent the Israeli physician population as a whole. For this reason, further study demonstrating results from other Israeli hospitals would be beneficial. Also, open-ended instead of multiple choice questions could overcome randomly correct answers of participating physicians and possibly be more informative in certain questions. Finally, nonresponse bias is another limitation of our study, since People who were “not available” during the meetings in which the questionnaire was administered may be different from people who were available at that time.

Considering that the study sample represented a population that serves most of the population of northern Israel and considering the low knowledge scores, encouraging similar studies in other hospitals would be useful for promoting transfusion medicine education in Israel. Based on our study results and on previous transfusion medicine assessments, we do not anticipate very different results.

In the framework of policy implications regarding improving physicians’ knowledge and thus reducing RBC transfusions, we believe that education in transfusion medicine must be increased, starting in medical school and continuing in designated educational programs, including periodic hospital-wide lectures and clinical scenarios discussed in group meetings. Also, using an electronic “pop-up” alert with each RBC transfusion order, containing data regarding relevant literature, such as the real-time clinical decision support used by the Stanford study, would be beneficial. The Israel Ministry of Health initiated an educational computer program for the medical staff regarding technical blood transfusion regulations. Nevertheless, these regulations were most recently updated in 2002 [27] and they do not replace the lack of RBC transfusion guidelines in Israel. A similar computerized educational program that focuses on indications for transfusion and guidelines in Israel could perhaps contribute to increasing physicians’ awareness. Implementation of such policy changes was associated with a total decrease in RBC utilization of approximately 40% in our hospital, similar to the potential reduction in RBC utilization discussed by the AABB [14]. Reducing RBC utilization can be translated into decreased patient morbidity and perhaps mortality. In their interventional monitoring program, Politsmakher et al. demonstrated a total decrease of 28.6% in the complication rate and 14% reduction in annual patient mortality [28]. Decreasing RBC utilization is also associated with substantial cost savings, as the price of each RBC unit is currently 230 ILS. Exploiting different platforms to increase physicians’ awareness is thus associated with improved patient safety and effective practice.

## Conclusions

According to the Choosing Wisely campaign which calls for limiting transfusions and lowering the transfusion threshold, a restrictive threshold (7.0–8.0 g/dl) should be used for most hospitalized, stable patients without evidence of inadequate tissue oxygenation. Our data suggest that there is a lack of general and basic knowledge in transfusion medicine among physicians in the non-operating room setting, which may play a role in RBC transfusion overuse. Personal background characteristics such as medical specialty and professional status may improve knowledge of transfusion medicine. Educational programs and increasing physicians’ awareness might help reduce unnecessary transfusions.

## Additional file

Additional file 1: Blood transfusion questionnaire. (DOCX 14 kb)

## Abbreviations

IQR: Interquartile range
RBC: Red Blood Cells
TRICC: Transfusion requirements in critical care
