# Supplementary material for: Physicians’ lack of knowledge - a possible reason for red blood cell transfusion overuse?
Source: Isr J Health Policy Res. 2017 Dec 12;6:49. doi: 10.1186/s13584-017-0173-0 (PMC5725969; doi:10.1186/s13584-017-0173-0)
Supplement: Additional file 1: — Blood transfusion questionnaire. (DOCX 14 kb) [file 13584_2017_173_MOESM1_ESM.docx]

Appendix

**Blood transfusion questionnaire** (correct answers are in bold font)

**A. Personal background**

1. Gender: (M / F)

2. Age (years): ________

3. Attended medical school in: Israel / Other _________

4. Number of years practicing medicine: _________

5. I am: a senior physician / a resident / an intern / house doc (on-duty nights only)

6. Field of medicine: ___________________

7. My unit of medicine is in: internal medicine wing / surgical wing

8. My family origin is (you may circle more than one): Israel / Former Soviet Union / Eastern Europe / Western Europe / North America / South America / Asia / North Africa / Other

**B. Professional section**

1. Have you heard of RESTRICTIVE BLOOD MANAGEMENT (RBM) policy? Yes / No If so, when? Where? ____________________________________________________________

2. Do you use any guidelines when you perform a blood transfusion? Yes / No

3. The logic behind the RBM policy is:

a. financial saving

b. **Patient benefit**

c. Both are correct

4. Do you know the Hemoglobin (Hb) threshold for an RBM blood transfusion? Yes / No

5. In LIBERAL BLOOD MANAGEMENT (LBM) policy the Hb threshold for blood transfusion is 10 g/dl. Was this determined by Evidence Based Medicine? Yes / **No**

6. Does the patient's medical status determine the decision to use RBM or LBM in blood transfusion? **Yes** / No

7. The only reason for giving a blood transfusion is to improve tissue oxygen delivery. **True** / **False**. If not true, then what are other reasons? ______________________________

8. What is the most exact parameter of oxygen delivery status?

a. Hemoglobin level

b. **Intracellular Po2**

c. Extracellular Po_2_

d. Hematocrit %

e. Don't know

9. At what % of acute blood loss should you begin transfusing blood?

a. 15%-20%

b. 20%-30%

c. **30%-40%**

d. 40%-50%

e. Don't know

10. The disadvantage of transfusing blood according to the restrictive policy vs. the liberal policy is the slightly higher mortality rate, though the cost efficiency is very high in the restrictive policy, so it is worthwhile. True / **False**

11. Gastrointestinal bleeding can be continuous, so giving a blood transfusion before Hb drops to less than 8 g/dl and with no accompanying symptoms, will greatly improve the outcome for these patients. True / **False**

12. In ischemic heart disease, one should not consider restrictive blood transfusion, and should keep Hb above 10 g/dl. True / **False**

13. In case of acute myocardial infarction, giving a blood transfusion at Hb threshold between 8-10 g/dl may harm the patient and, generally, increases mortality. True / **False**

14. A patient who has undergone orthopedic surgery (such as THR) should have an Hb level of more than 10 g/dl in order to be able to undergo physiotherapy. True / **False**

15. A blood transfusion in a patient hospitalized in the ICU with acute blood loss will prevent mortality within 30 days:

a. In a patient with Hb = 10 g/dl

b. In a patient with Hb = 7-8 g/dl

c. **There is no difference between Hb levels in preventing mortality**

d. Don't know

16. Which of the following is not an immediate complication of blood transfusion?

a. Fever

b. Allergic reaction

c. **GVHD**

d. Circulatory overload

e. Acute respiratory damage

17. Current transfusion guidelines do not support the RBM policy and continue to recommend transfusing blood according to Hb level <10 g/dl, without consideration of other parameters (i.e. clinical symptoms). True / **False**

18. In general, there is a lack of clear guidelines which leads to confusion among physicians regarding the field of transfusion medicine. True /**False**

19. RBM is a modern policy which has been adopted by only a few U.S. hospitals. Until it is accepted in Europe and Australia, conclusions regarding this policy cannot be done. True / **False**

20. If cost efficiency were not so important in healthcare, the RBM policy would not be implemented. True / **False**
